# Biopolymers in Drug and Gene Delivery Systems

**DOI:** 10.3390/ijms241612763

**Published:** 2023-08-14

**Authors:** Yury A. Skorik

**Affiliations:** Institute of Macromolecular Compounds of the Russian Academy of Sciences, Bolshoi VO 31, St. Petersburg 199004, Russia; yury_skorik@mail.ru

Recent years have seen remarkable advances in the field of drug and gene delivery systems, revolutionizing the way we approach therapeutic treatments. The design and development of these systems has become increasingly important because of their potential to improve the efficacy and safety of drug and gene therapies and to overcome several challenges faced by traditional therapeutic approaches. Many drugs have limited stability, poor solubility, or face barriers in reaching their intended targets in the body. The development of delivery systems that can encapsulate these therapeutic agents and efficiently transport them to the desired location can greatly improve their efficacy. In addition, such systems can protect the agents from degradation, increase their bioavailability, and provide controlled release, allowing for prolonged therapeutic effects. In addition, targeting specific cells or tissues with therapeutic agents is a critical aspect of personalized medicine.

Drug and gene delivery systems with targeting capabilities, such as ligand–receptor interactions or surface modifications, can selectively deliver therapeutic agents to specific cells or tissues, maximizing their effects and minimizing off-target toxicity. This targeted delivery approach holds great promise for the treatment of complex diseases such as cancer, where precision and specificity are essential. In addition to targeted delivery, the design and development of drug and gene delivery systems also play a critical role in improving the stability and pharmacokinetics of therapeutic agents. Many drugs are rapidly metabolized or cleared from the body, limiting their efficacy. However, by formulating these agents in appropriate delivery systems, their stability can be improved, and their release can be controlled, ensuring a sustained and prolonged effect.

This Special Issue aims to emphasize the significance of designing and developing drug and gene delivery systems based on biopolymers and their potential applications, as well as their impact on the future of medical treatment. When we refer to biopolymers, we are referring to a range of polymers, not only natural polymers produced by living organisms, but also semi-synthetic polymers (i.e., modified natural polymers) and synthetic polymers that are biocompatible, biodegradable, and capable of creating drug delivery systems. Both natural and synthetic biopolymers have their respective advantages and disadvantages. Natural biopolymers are preferred over synthetic biopolymers due to their biocompatibility, biodegradability, and environmental safety. Synthetic biopolymers, on the other hand, have distinctive advantages in terms of stability and adaptability to various biomedical applications.

The Special Issue contains eleven articles, including nine original research papers and two reviews. The authors come from different geographical locations, including North America (USA and Mexico), Europe (UK, Germany, Sweden, and Russia), and Asia (China, Saudi Arabia, Pakistan, Malaysia, and Singapore). The papers cover various topics related to the use of biopolymers (polysaccharides such as cellulose and its derivatives, starch and its derivatives, chitosan and its derivatives, hyaluronan, sodium alginate, agarose, arabinoxylan, heparin, cyclodextrin; peptide and proteins; as well as synthetic biopolymers) for the delivery of various drugs and therapeutic nucleic acids. The types of biomaterials studied include polymer conjugates, nano- and submicroparticles, gels, films, and implants for drug administration via intravascular, intravitreal, buccal, topical, and implantation routes.

The reviews of the Special Issue focus on the use of cellulose [1] and cyclodextrin (CD) [2] in the development of gels for various biomedical applications. The review by Tyshkunova et al. [1] discusses the structure of cellulose and its properties as a biomaterial, the strategies for dissolving cellulose, and the factors that influence the structure and properties of the resulting cryogels. It focuses on the advantages of the freeze-drying process and highlights recent studies on the preparation and application of cellulose cryogels in wound healing, the regeneration of various tissues (e.g., damaged cartilage, bone tissue, and nerves), and controlled release drug delivery. Liu et al. in their review [2] discuss various methods for the design and preparation of CD hydrogels and summarize the potential applications of drug-loaded CD hydrogels. As a natural oligosaccharide, CD has shown remarkable application prospects in hydrogel development. CD can be incorporated into hydrogels to form chemically or physically cross-linked networks. The unique cavity structure of CD makes it an ideal vehicle for drug delivery to target tissues. This review broadens our understanding of the development trends in the application of CD-containing hydrogels, which lays the foundation for future clinical research.

Several strategies have been explored to develop stimulus-responsive hydrogels for designing smart drug delivery platforms that can release drugs at specific targeted areas and at predetermined rates. However, few studies have investigated how innate hydrogel properties can be optimized and modulated to tailor drug dosage and release rates. Briggs et al. [3] investigated the individual and combined effects of polymer concentration and crosslinking density (controlled by chemical crosslinking with N,N′-methylenebis-acrylamide and physical crosslinking with silica nanoparticles) on drug delivery rates, using polyacrylamide gel and 5-fluorouracil as a model system. The experiments showed a strong correlation between hydrogel properties and drug release rates and demonstrated the existence of a saturation point in the ability to control drug release rates using a combination of chemical and physical crosslinkers. This study provides a basis for developing tunable hydrogel platforms, including polymeric nanocarriers and nanogels, for delivering a wide range of therapeutics.

Two of the original papers in this Special Issue focus on the development of delivery systems for peptide antibiotics polymyxin B (PMXB) and E (PMXE) [4,5]. In recent years, unsuccessful treatments of multidrug-resistant bacterial infections have caused previously dismissed antibiotics to resurface. One such group of antibiotics is the polymyxins (PMXs)—cyclic peptide antibiotics that primarily target Gram-negative pathogens. Although this group of antibiotics was introduced into clinical practice more than 50 years ago, it was soon discovered to have serious side effects such as nephro- and neurotoxicity. Nevertheless, the World Health Organization reclassified PMXs as antibiotics that are critically important for treating infections with few or no alternative options. The reintroduction of PMXs as therapeutic agents in clinical practice has spurred the search for methods to reduce their side effects [6]. A nano-sized PMXE delivery system with hydrodynamic diameters (D_h_) of 210–250 nm and a ζ-potential of −19 mV has been proposed by Dubashynskaya et al. [4] for intravascular injection. This delivery system is based on a polyelectrolyte complex between hyaluronate (polyanion), diethylaminoethylchitosan (polycation), and positively charged PMXE. In vitro experiments demonstrated that both encapsulated and pure PMXE had a minimum inhibitory concentration of 1 μg/mL against *Pseudomonas aeruginosa*, indicating that encapsulating PMXE in polysaccharide carriers does not reduce its antimicrobial activity. A hybrid delivery system of core–shell nanoparticles (D_h_ of about 100 nm) has been proposed by Iudin et al. [5]. These hybrid nanoparticles consist of an Ag core and a poly(glutamic acid) shell that can bind PMX via electrostatic interactions. The hybrid nanoparticles showed no cytotoxicity, had low macrophage uptake, and demonstrated intrinsic antimicrobial activity. Furthermore, composite materials based on agarose hydrogel were developed, comprising both the PMX-loaded hybrid nanoparticles and free PMX (PMXB or PMXE). The antibacterial activity of PMX-loaded hybrid nanoparticles and composite gels against *P. aeruginosa* was evaluated, and the results showed that the PMX hybrid delivery system had a synergistic effect compared to either the antibiotics or Ag nanoparticles.

The development of biopolymer composite films for drug delivery is the subject of three papers in the Special Issue [7,8,9]. Lim et al. [7] evaluated the feasibility of using three mucoadhesive polysaccharides (hydroxypropyl methylcellulose, starch, and hydroxypropyl starch) to develop curcumin-loaded buccal films delivered in the form of chitosan nanoparticles. The results indicated that hydroxypropyl starch is the most suitable mucoadhesive polysaccharide for developing curcumin-loaded buccal films due to the superior curcumin release, good payload uniformity, minimal weight/thickness variations, high folding resistance, and good long-term storage stability of the composite films. Alzarea et al. [8] fabricated and characterized films composed of arabinoxylan (from *Plantago ovata*) and sodium alginate (from brown algae) loaded with gentamicin sulfate, an aminoglycoside antibiotic, for potential use as wound dressings. These films displayed excellent antibacterial effects and desirable properties for wound dressings, such as a tensile strength similar to human skin, mild capacity for water/exudate uptake, suitable water transmission rate, and excellent cytocompatibility. López-Saucedo et al. [9] used an alternative approach to develop antibacterial films. This was achieved via the surface modification of polypropylene films using gamma-irradiation-induced grafting with methyl methacrylate and N-vinylimidazole, which provided a suitable surface capable of loading vancomycin, a glycopeptide antibiotic. The composite multilayer film surface exhibited moderate hydrophilicity and pH-responsiveness, properties desirable for controlled drug release.

Another example of surface modification is presented in the paper by Facchetti et al. [10], where the authors proposed a simple and convenient way to modify the surface of titanium alloy (Ti6Al4V) bone implants with biopolymers consisting of whey protein isolate (WPI) fibrils and heparin or tinzaparin (low molecular weight heparin) to enhance the proliferation and differentiation of bone-forming cells. The results showed that WPI fibrils are an excellent material for biomedical coatings because they are easily modifiable and resistant to heat treatment. In addition, a heparin-enriched WPI coating improved the differentiation of human bone marrow stromal cells by increasing tissue non-specific alkaline phosphatase activity.

The development of delivery systems for the intravitreal administration of glucocorticoids for the treatment of inflammatory conditions in the posterior segment of the eye is challenging. Intravitreal delivery systems have the potential to provide several advantages, such as overcoming anatomical and physiological barriers, increasing bioavailability, and prolonging and regulating drug release over several months. The conjugation of glucocorticoids with biopolymers is a viable approach for the development of intravitreal drug delivery systems, as it prevents rapid elimination and provides targeted and controlled drug release [11,12]. Dubashinskaya et al. [13] demonstrated the potential feasibility of this approach using a dexamethasone conjugate with succinyl chitosan. The developed conjugates showed sustained and prolonged (over one month) release of dexamethasone and significant anti-inflammatory effects in TNFα-induced and LPS-induced inflammation models, suppressing CD54 expression in THP-1 cells by 2- and 4-fold, respectively. These novel conjugates of succinyl chitosan and dexamethasone show promise as ophthalmic carriers for intravitreal administration.

A promising polymeric gene delivery system based on cysteine-flanked arginine-rich peptides modified with a cyclic RGD moiety was proposed by Egorova et al. [14]. The system is designed for targeted DNA delivery to uterine fibroid cells. The carrier can form small (D_h_ of 100–200 nm) and stable polyplexes that effectively protect DNA from nuclease degradation. The specificity of DNA delivery to αvβ3 integrin-expressing cells was confirmed by cell transfection experiments, which showed a 3-fold increase in transfection efficiency as a result of the RGD modification. Primary cells obtained from myomatous nodes of uterine leiomyoma patients were used to model suicide gene therapy by transferring the HSV-TK suicide gene, resulting in a 2.3-fold decrease in proliferative activity after ganciclovir treatment of the transfected cells. Pro- and anti-apoptotic gene expression analysis confirmed that the polyplexes stimulate uterine fibroid cell death in a suicide-specific manner. Thus, this peptide carrier can be used in further efforts to develop uterine leiomyoma suicide gene therapy.

In summary, the design and development of drug and gene delivery systems is of paramount importance in the field of therapeutics. These systems have the potential to overcome various challenges associated with traditional therapeutic approaches, improve drug and gene stability, enable targeted delivery, and enhance therapeutic efficacy. With ongoing advances in nanotechnology and biomaterials research, these systems are poised to revolutionize the way we approach medical treatments, opening up new possibilities for personalized and effective therapies.
